# Tyrosine Sulfation at Antibody Light Chain CDR-1 Increases Binding Affinity and Neutralization Potency to Interleukine-4

**DOI:** 10.3390/ijms25031931

**Published:** 2024-02-05

**Authors:** Aaron M. D’Antona, Julie M. Lee, Melvin Zhang, Clarence Friedman, Tao He, Lidia Mosyak, Eric Bennett, Laura Lin, Maddison Silverman, Funi Cometa, Caryl Meade, Tyler Hageman, Eric Sousa, Justin Cohen, Kimberly Marquette, Darren Ferguson, Xiaotian Zhong

**Affiliations:** 1 BioMedicine Design, Pfizer Research & Development, 610 Main Street, Cambridge, MA 02139, USAtaohe99@yahoo.com (T.H.); tyler.hageman@pfizer.com (T.H.); eric.sousa@pfizer.com (E.S.);; 2 Translational Clinical Sciences, Pfizer Discovery & Early Development, 610 Main Street, Cambridge, MA 02139, USA; 3 Inflammation and Immunology Research Unit, Pfizer Research & Development, 610 Main Street, Cambridge, MA 02139, USA

**Keywords:** tyrosine sulfation, complementarity-determining region (CDR), antigen-binding affinity, interleukine-4, cytokine signaling

## Abstract

Structure and function of therapeutic antibodies can be modulated by a variety of post-translational modifications (PTM). Tyrosine (Tyr) sulfation is a type of negatively charged PTM that occurs during protein trafficking through the Golgi. In this study, we discovered that an anti-interleukin (IL)-4 human IgG1, produced by transiently transfected HEK293 cells, contained a fraction of unusual negatively charged species. Interestingly, the isolated acidic species exhibited a two-fold higher affinity to IL-4 and a nearly four-fold higher potency compared to the main species. Mass spectrometry (MS) showed the isolated acidic species possessed an +80-Dalton from the expected mass, suggesting an occurrence of Tyr sulfation. Consistent with this hypothesis, we show the ability to control the acidic species during transient expression with the addition of Tyr sulfation inhibitor sodium chlorate or, conversely, enriched the acidic species from 30% to 92% of the total antibody protein when the IL-4 IgG was co-transfected with tyrosylprotein sulfotransferase genes. Further MS and mutagenesis analysis identified a Tyr residue at the light chain complementarity-determining region-1 (CDRL-1), which was sulfated specifically. These results together have demonstrated for the first time that Tyr sulfation at CDRL-1 could modulate antibody binding affinity and potency to a human immune cytokine.

## 1. Introduction

Post-translational modifications (PTMs) in mammalian cells provide de novo synthesized proteins with an additional level of structural diversity beyond their primary sequences [1,2,3,4]. These PTMs impose new biological functions and activity modulations as well as further physiological consequences [3,4,5,6,7]. For the discovery and development of protein therapeutics, PTMs are critical quality attributes for final clinical products, and the dependence on the biological systems for producing intended PTMs is a unique challenge for biologics drugs. Tremendous efforts in protein engineering and process development have also uncovered new findings in PTMs. Investigating and understanding the discoveries in PTMs for protein therapeutics can not only improve product quality but also potentially bring out new applications in molecular design and product production.

Tyrosine (Tyr) sulfation is a common type of PTM for secreted proteins or membrane proteins, in which a negatively charged sulfate group from 3′-phosphoadenosine 5′-phosphosulfate is transferred to the hydroxyl group of a Tyr residue through an O-sulfate ester [8,9,10]. This modification in mammalian cells is catalyzed by two Golgi membrane-bound tyrosylprotein sulfotransferases (TPST-1 and TPST-2), which share 64% sequence homology [11,12,13,14]. They have different substrate specificities [15,16,17] and distinct mouse knockout phenotypes [18,19]. The TPST enzymes are conserved from worm to mammals [8], but structurally distinct from those in plants [20] and some prokaryotes [21], suggesting a wider role of this PTM across many domains of life [22,23]. Tyr sulfation has been shown to be involved in many biological processes, including blood coagulation, leukocyte rolling, complement cascade, hormonal regulation, chemokine signaling, collagen binding, and natural humoral immune response to viral infection, as well as plant cell proliferation [8,10,24,25,26].

Tyr sulfation has also been observed for a handful of recombinant therapeutic antibodies such as immunoglobulin G (IgG) [25,26,27,28,29,30,31], which represents a major class of biologics for disease treatments [32,33,34,35]. This PTM is located in the antibody fragment antigen-binding domain (Fab), which forms a contact with antigens through six regions of sequence variability termed the complementarity-determining regions (CDRs) [36,37]. For several anti-human immunodeficiency virus type-1 (HIV-1) antibodies, e.g., PG16 and 412d [31,38], tyrosine sulfation at the CDRH3 of antibody heavy chain (HC) can lead to an increase in affinity for the target protein gp120 by 4–500-fold [39,40,41]. However, tyrosine sulfation in some antibodies has no impact on the antigen binding [27,28,29], and the biological function for this PTM in therapeutic antibodies remains unclear.

Interleukin-4 (IL-4) is a secreted cytokine involved in the induction and persistence of the type 2 immune response [42], which is believed to be associated with atopic diseases such as asthma and atopic dermatitis [43]. In this study, we produced anti-IL-4 human monoclonal antibodies by transient transfection in HEK293 cells for in vitro and in vivo investigation. Unexpectedly, one anti-IL-4 antibody 3B9 was found containing two unusual negatively charged acidic species in an anion-exchange chromatographic analysis. Mass spectrometry (MS) analysis revealed that they contained an additional mass of +80 Da in its light chain, suggesting that they could be sulfate-modified at a Tyr residue. Consistently, the acidic species completely disappeared when the cell culture was treated with sodium chlorate, the specific inhibitor for tyrosine sulfation [44]. When TPST-1 and -2 were co-transfected during HEK293 transient expression with the 3B9 antibody, the acidic species were drastically enriched. Interestingly, the analysis of Biacore binding to IL-4 and STAT6 phosphorylation assay showed that the acidic species had a higher affinity to IL-4 and were more potent in cell-based signaling assays than the control species. Further MS and mutagenesis analysis indicated that a Tyr residue at the light chain CDR-1 was specifically sulfated.

## 2. Results

### 2.1. Identification of Unusual Negatively Charged Acidic Species in Anti-IL-4 3B9 Antibody Produced by Transient Transfection in HEK293 Cells

**Figure 1:**
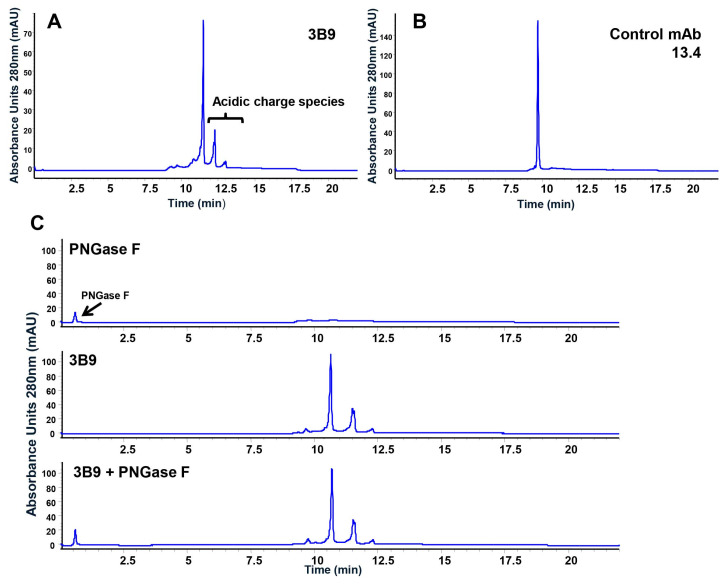
Strong SAX analysis reveals unusual negatively charged acidic species in anti-IL-4 3B9 antibody. SAX chromatographic analysis of anti-IL-4 antibodies was performed as described in Section 4. (**A**) SAX chromatographic analysis of antibody 3B9. (**B**) SAX chromatographic analysis of control antibody 13.4. (**C**) SAX chromatographic analysis of 3B9 digested with PNGaseF.

We produced several human IgG1 monoclonal antibodies with high affinity against IL-4 in a transient HEK293 system for in vitro affinity-binding and cell-based signaling assays. When purified antibody proteins were subjected to various chromatographic analyses for quality control prior to product release, strong anion-exchange chromatographic (SAX) analysis revealed two negatively charged acidic species in the antibody 3B9 (Figure 1A) totaling ~37% of the total area under the curve (AUC). These acidic species were not detectable in other antibodies such as 13.4 (Figure 1B). Analytical size-exclusion chromatography and SDS-PAGE analysis confirms the 3B9 antibody to be intact. Analytical hydrophobic interaction chromatography (HIC) analysis of 3B9 shows similar profiles to those of the antibody 13.4. The unusual acidic species detected in the antibody 3B9 suggest a form of PTM occurred in the protein molecule. Treatment of the 3B9 sample with PNGase F showed no change in the SAX charge-speciation (Figure 1C).

**Figure 2:**
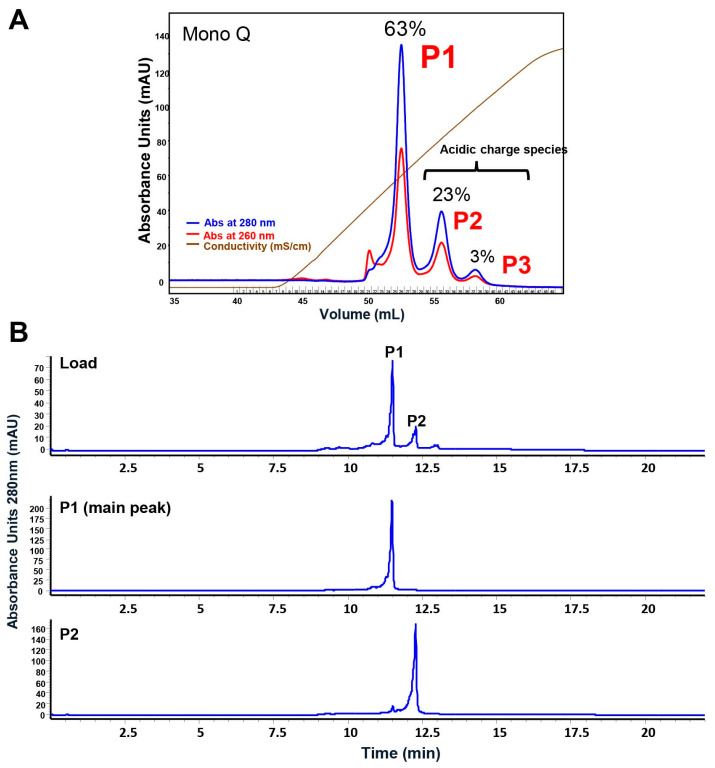
Preparative anion-exchange purification on 3B9 antibody. Preparative-scale anion-exchange Mono Q purification of 3B9 was performed as described in Section 4. (**A**) Chromatographic data of preparative-scale anion-exchange purification of 3B9. (**B**) Analytical SAX analysis of the Mono Q load, P1, and P2 from the preparative samples.

To further characterize these species, additional purification steps were taken on the 3B9 protein materials. Preparative-scale separation of 3B9 using a Mono Q column resulted in a similar profile observed with the analytical SAX method with good separation of acidic species (Figure 2A). Fractions containing the first acidic species (P2, ~23% of the total population) and main peak (P1, ~63% of the total population) were isolated. The second acidic species (P3) was not isolated. It totaled ~3% of the total recovered protein and was too low to collect. Fractions isolated by preparative Mono Q containing P1 and P2 we reapplied to the SAX analysis to confirm isolation and purity of each peak (Figure 2B). Accordingly, these two peak fractions were subjected to further analyses.

### 2.2. The Acidic Species of 3B9 Antibody Exhibited a Higher Binding Affinity and Neutralization Potency to IL-4 Than the Main Species

**Figure 3:**
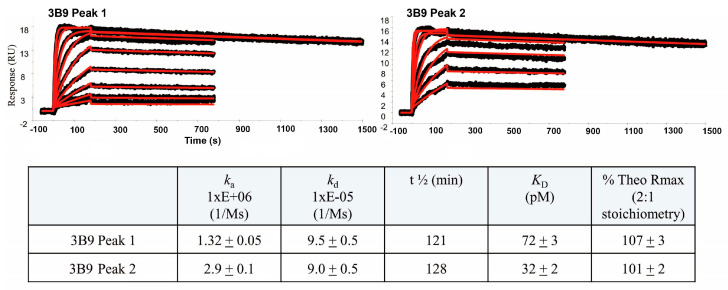
Biacore analysis indicates that the acidic species of 3B9-P1 antibody exhibits a two-fold higher binding affinity to IL-4 than the main species 3B9-P2. The acidic species of 3B9 or the main species of 3B9 were captured onto directly immobilized anti-human IgG on a Biacore sensor chip CM5 surface on a Biacore T200 instrument. Human IL-4 with various concentrations were injected and analyzed as described in Section 4. All data were fit to a 1:1 Langmuir binding model in Biacore T200 Evaluation software v 2.0.1 (fit lines shown in red). Data shown are representative of six surfaces between two independent experiments. Kinetic constants are shown in the table. IL = interleukin; KD = dissociation constant; RU = response units.

To ascertain whether there is any activity difference between the main peak [3B9-Peak1 (P1)] and the acidic species [3B9-Peak2 (P2)] in terms of binding affinity, a Biacore binding to human IL-4 (hIL-4) was performed (Figure 3). To achieve this, 3B9-P1 or 3B9-P2 were captured onto directly immobilized anti-human IgG on a Biacore sensor chip CM5 surface on a BiacoreT200 instrument. hIL-4 with various concentrations were injected and allowed to associate at 25 °C. hIL-4 bound with a faster on rate to the acidic 3B9-P2 than to the main peak 3B9-P1. The KD of the acidic 3B9-P2 antibody was two-fold lower than that of the main peak 3B9-P1 antibody (32 pM vs. 72 pM). The acidic 3B9-P2 had a faster association constant than the main peak 3B9-P1 (2.9 × 10^6^ vs. 1.32 × 10^6^) whereas their disassociation constants were similar (9.0 × 10^5^ vs. 9.5 × 10^5^). These results indicate that the acidic species possesses a higher affinity to human IL-4 than the main species.

**Figure 4:**
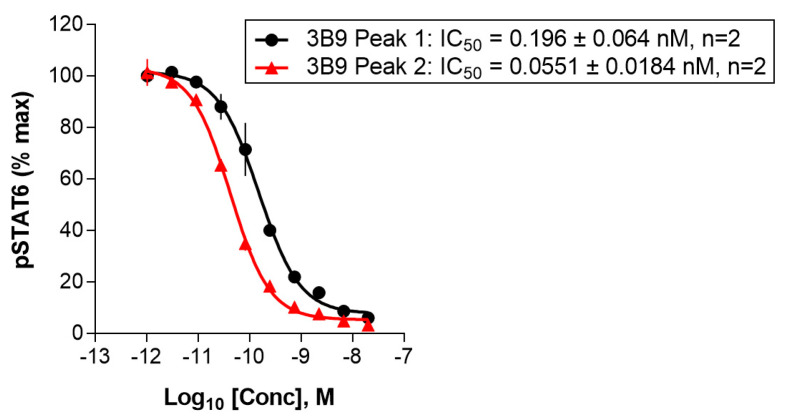
The STAT6 phosphorylation HT29 cell-based assay indicated that the acidic species had 3.6-fold higher potency than the main species. HT29 pSTAT6 assay was performed as described in Section 4.

To find out whether there is any activity difference between the main peak (3B9-P1) and the acidic species (3B9-P2) in neutralization potency, a HT29 pSTAT6 assay of IL-4 as described in Section 4 was performed. Both protein species of 3B9 exhibit potent anti-IL-4 neutralization efficacy. As shown in Figure 4, the acidic species (3B9-P2) had 3.6-fold lower IC50 in HT29 pSTAT6 assay than that of the main peak 3B9-P1 (0.055 nM vs. 0.196 nM). These data indicate that the acidic species is more potent than the main species in neutralizing IL-4.

### 2.3. The Acidic Species of the 3B9 Antibody Contained a Sulfated Tyrosine on the CDR1 of Its Light Chain

**Figure 5:**
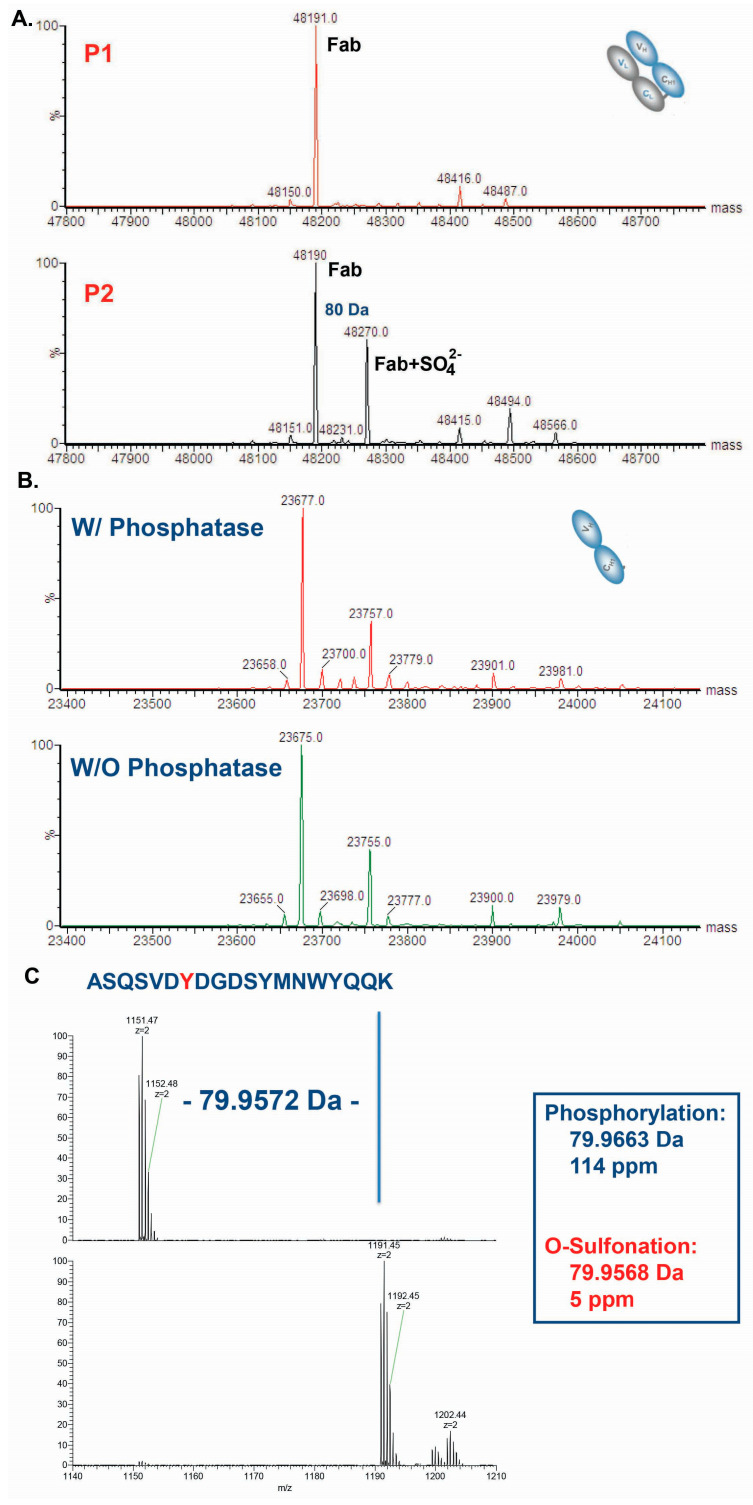
The MS analysis on Fab of the 3B9 antibody indicates that an 80Da mass increase in its light chain was due to an O-sulfation in a Tyr residue. Then, 3B9-P1 and 3B-P2 were digested with LysC to generate Fabs and subjected to the LC–MS analysis (Panel (**A**)) performed as described in Section 4. (**B**) The 3B9-P2 samples either treated with (W/) or without (W/O) alkaline phosphatase, were subjected to LC–MS analysis. (**C**) The accurate mass increase for a short peptide around CDRL1 region further confirmed that the PTM was O-sulfation.

Since the acidic species of 3B9 might be a form of PTM to the main species, LC–MS analysis was performed. To achieve this, 3B9-P1 and 3B9-P2 antibody species were digested with protease LysC to generate Fc (fragment crystallizable) and Fab. The masses of Fc and HC in both species were identical, whereas the 3B9-P2 contained an additional LC species with a delta mass increase of 80 Da from the expected mass (Figure 5A). The mass increase suggests the modification of sulfation or phosphate. As shown in Figure 5B, the treatment with alkaline phosphatase did not abolish the +80 Da mass increase, indicating that the PTM was not phosphorylation. As shown in Figure 5C, the accurate mass increase (79.9572) matched well with the mass of O-sulfation (79.9568), further confirming that a sulfation at a tyrosine residue occurred in the light chain of 3B9.

**Figure 6:**
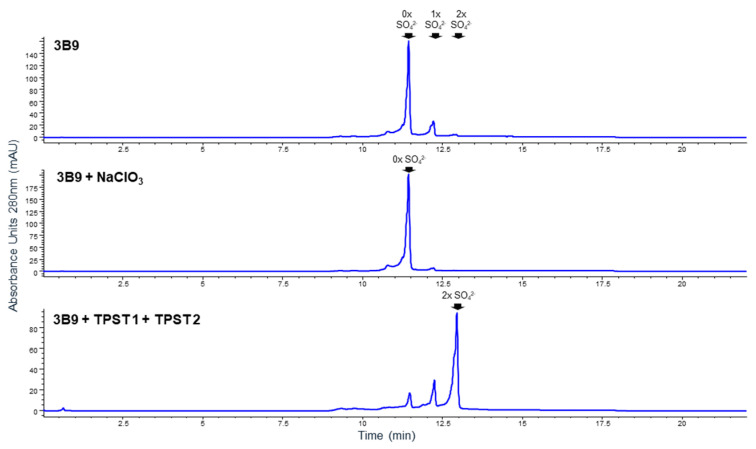
Sodium chlorate treatment eliminated acidic species and overexpressing TSPT1- and 2-enriched acidic species. A total of 30 mM of sodium chlorate was added to the cell culture 16 h post-transfection. TPST-1 and TPST-2 genes were co-transfected with 3B9 HC and LC DNAs in the ratio of 1:1:1:1. Conditioned medium was harvested on day 5, purified, and analyzed as described in Section 4.

To confirm this hypothesis, transient production of 3B9 in HEK293 cells was treated with sodium chlorate, the inhibitor for protein sulfation in intact cells [44]. As shown in Figure 6, the acidic species of 3B9 nearly disappeared in a concentration-dependent manner (from 30% without NaClO_3_ treatment to 2.6% at 30 mM NaClO_3_). This result indicates that the reduction of the acidic species correlates with the inhibition on sulfation process by sodium chlorate. Interestingly, when TPST-1 and TPST-2 genes were co-transfected during HEK293 transient expression along with the 3B9 antibody, the main species was drastically reduced and the P3 species were drastically enriched. The total fraction of the acidic species [P2 (1xSO_4_^−^) and P3 (2xSO_4_^2−^)] was 92%. The IC50 value of these enriched species in the neutralization pSTAT6 assay of IL-4 correlated well with 3B9-P2 and P3 (Table 1). These data indicate that these Tyr–sulfate enzymes increase the modification of the 3B9 antibody.

**Figure 7:**
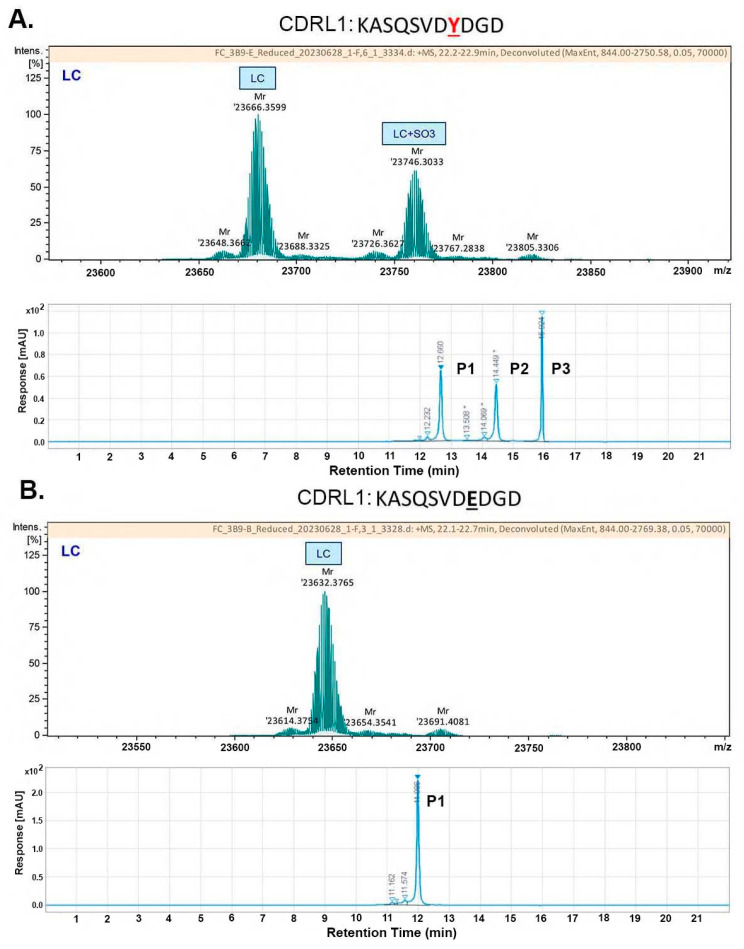
Tyr 31 (Y31, L27D) residue at the light chain CDR1 of antibody 3B9 is sulfated as demonstrated by Y31E mutation in the MS analysis and the SAX profiling. (**A**) The MS analysis and the SAX profile of 3B9 co-transfected with TPST-1/2. (**B**) The MS analysis and the SAX profile of 3B9 Y31E mutant co-transfected with TPST-1/2.

**Figure 8:**
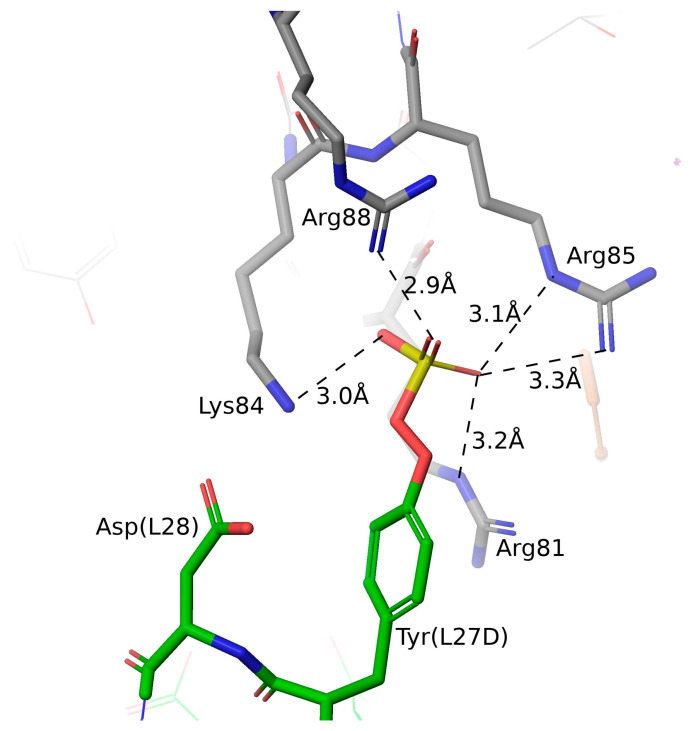
Structure modeling of the interactions between the sulfate and a basic patch on IL-4. IL-4 is shown with gray carbons, and the antibody light chain with green carbons. Sulfation was modeled onto the Tyr31 (L27D) side chain, and its behavior was monitored during a 250 ns molecular dynamics simulation, with Desmond release 2023-3 (Schrodinger, LLC., New York, NY, USA). Electrostatic interactions (<5.0 Å distance between sulfate S and guanidinium C) were observed in more than half of the frames for Arg81 of IL-4, with lower frequency for Arg85 and Arg88. Interaction with Lys84 was less frequent, although some frames, including the one shown here from halfway through the simulation, show interactions between the sulfate and all four side chains.

To further verify the Tyr-sulfation modification, LCMS and sequence analysis was performed and revealed a delta mass consistent with a sulfation on the LC (Figure 7A) and that the Tyr (Y31, L27D) at LC CDRL1 (KASQSVDYDGD) met the criteria for Tyr sulfation site [45]. Replacing this Tyr with Asp eliminated the acidic species even when TPST-1/2 genes were co-transfected (Figure 7B). Consistently, the acidic species in the SAX analysis (P2 and P3) also disappeared, with only unsulfated P1 present in the 3B9 Y31E variant (Figure 7B vs. Figure 7A). These data together demonstrate that Y31 at CDRL1 was the sulfated residue and was attributed to the acidic species detected in the SAX analysis. Structural modeling analysis of 3B9 suggests that the sulfate at the CDRL-1 might interact with a basic patch on human IL-4 (Figure 8).

## 3. Discussion

In this study, we discovered two unusual negatively charged acidic species for an anti-IL-4 antibody 3B9 during the SAX analysis. These acidic species were shown to possess a higher affinity and potency to IL-4 than those of the main species. Further investigation revealed that the acidic species were a result of sulfation modification to a Tyr residue in the CDR-1 of the light chain. To the best of our knowledge, this is the first example in which Tyr sulfation on an antibody’s CDRL1 regulated its binding affinity and potency. During immunoglobulin rearrangement, CDRL1 (with only V gene fragments) is known to be much less diverse than CDRL3 (with additional J fragment recombination) or CDRH3 (with additional D or J recombination). Our findings indicate that a PTM like Tyr sulfation can provide an additional diversification mechanism for modulating antibody’s binding affinity and specificity, beyond the gene recombination processes such as V (D)J recombination and somatic hypermutation.

Tyr is a bulky amino acid that can facilitate hydrophobic and pi stacking interactions, and is, therefore, frequently found in the CDR regions of antibodies. Although the rigid sequences for the sulfotransfer modification are still not fully defined [45,46,47,48,49,50], the presence of acidic residues flanking a potential acceptor tyrosine [23,51,52] could be presumably found in many of the CDR loops. This would make them a possible substrate for the TPST enzymes, especially considering the CDRs as the loop structures where the tyrosine sulfation modification might tend to occur [23,49]. However, thus far, only a handful of antibodies have been reported with the tyrosine sulfation modification [26,31]. Notably, eight anti-HIV-1 antibodies targeting the Env protein gp120, e.g., 412d, E51, PG9, PG16, PGT145, 2909, CAP256.03, and CAP256.25, were found to be Tyr-sulfated at the CDRH3 regions [25,29,30,40,53]. Further studies [39,40,41] reveal that increased levels of tyrosine sulfation in these anti-HIV-1 antibodies corresponds to much more robust binding to the V1V2 domain of the Env protein antigen. These publications demonstrate that the Tyr sulfation at the antibody CDRs can modulate antibody affinity, serving as a strategy for natural humoral immune response to viral infection [25]. Tyr sulfation also occurs at the antibody CDRL1 [27] and CDRL2 [28] regions, though the corresponding antibody -binding affinities are not affected. The results from this study not only provide another example of Tyr-sulfated antibody at the CDRL1 region, but also reveal that this modification at this light chain region can also help establish a stronger intermolecular interaction between the antibody and the antigen. In addition, different from the examples of those anti-viral envelope protein antibodies with the modification at the long CDRH3 region, anti-IL-4 antibody 3B9 with the modification is in the short CDRL1 region targeting a cytokine protein.

The mechanism for the affinity increases by Tyr sulfation at the antibody CDRs likely involves the charge–charge interaction between the negative charges of sulfates and positive charges of the residues in the antigens. For anti-HIV-1 gp120 antibody, the Tyr-sulfated antibodies engage with the positively charged residues of gp120 in a manner like those of Tyr-sulfated chemokine receptors such as CCR5 [54,55,56]. Interestingly, IL-4 receptors are not Tyr-sulfated, but IL-4 contains basic patches on its protein surface. Charge–charge interaction between the sulfate group and Lys/Arg residues on IL-4 might explain the up-regulation on antibody affinity of Tyr-sulfated species. Our data support the previous hypothesis that humoral immune response could utilize sulfated tyrosine for selection for the antibody’s ability to bind antigens efficiently [25,26].

A sulfate group covalently attached to tyrosine is known to be acid labile and thermosensitive, which is also frequently lost, caused by the high-energy ionization and collision activation during MS analysis [23,51,52]. Therefore, it has been hypothesized that these features are the main reasons why the detection of tyrosulfo-modified proteins is much lower than estimated. Considering the wide presence of Tyr residues in the CDRs, a considerable number of Tyr-sulfated antibodies could also possibly exist without being detected. While ^35^S-sulfate labeling method, MS analysis, and Edman sequencing are reliable analytical tools for detecting Tyr sulfation, the SAX analytical method developed in the study, along with the chlorate treatment and the co-transfection of TPSTs, has established another quick way to identify tyrosine sulfation modification. While new MS methodologies are being developed for overcoming the current limitations [23,51,52], high-throughput methods such as immunodetection-based strategies [57,58] should be fully developed and validated for a rapid identification of tyrosine-sulfated proteins.

Tyr sulfation at CDRs has been previously reported to be part of the endogenous strategies for antibody diversification in the human immune repertoire in the scenario of HIV infection [25]. Since many recombinant therapeutic antibodies are derived from immune libraries of rodents or human, Tyr-sulfated antibodies can likely be detected from these semi-naturally occurring antibodies. In this report, the Tyr-sulfated antibody at CDRL1 was found to have a higher affinity to IL4, suggesting that endogenous autoreactive antibodies could potentially utilize this PTM as a mechanism for the increasing binding to those positively charged cytokines or receptors.

In addition, it is of interest to point out that Tyr sulfation also occurs in the Fc region of the antibody [59,60], other than the CDRs. Baeuerle and Huttner discovered that deglycosylated mouse IgG2a, a result from tunicamycin treatment to the hybridoma cells, was Tyr-sulfated in the Fc region near the N-glycosylation site [59]. Through site-directed mutagenesis and mass spec analysis, Masuda et al. reported that Tyr296 located in the Fc loop region of mouse IgG2b could be specifically sulfated when Asn297 was mutated to Ala [60]. The -1 position of Tyr296 is a negatively charged Asp residue, which makes it a potential substrate site for TPSTs when the masking N-glycans no longer exist [60]. These studies raise the possibility that protein engineering in the regions near surface-exposed Tyr residues could potentially introduce unexpected Tyr sulfation modification. The introduced negative-change and hydrophobic ring structure could possibly affect protein folding or function.

In summary, this study has provided an additional example of biophysiological function of Tyr sulfation. Other than the destabilization effects on Tyr-sulfated proteins such as sFRP-1 [61], this PTM can modulate antigen interaction. Similar to N-linked glycan at CDRs that enhance antigen binding [62,63,64], Tyr sulfation is another type of PTMs that have a role in regulating humoral immune responses and contributing to the extent of the antibody diversification [65]. Our data show that overexpressing the TSPT-1 and 2 genes can enrich Tyr-sulfated species, which could potentially make it therapeutically applicable through cell line engineering in mammalian cells or in plants [66].

## 4. Materials and Methods

### 4.1. Cell Culture and Transient Transfections

Mammalian HEK293F and Expi293 cells were cultured in an incubator with 5% CO2 at 37 °C in FreeStyleTM 293 medium or EXP293TM medium (Thermo Fisher Scientific, Waltham, MA, USA). Polyethylenimine (PEI)-based [67,68] or ExpifectamineTM-based [68,69] transient transfection process was used for antibody production. Briefly, plasmid DNAs encoding heavy chain and light chain of antibodies in the ratio of 1:1 at total 1 mg/L were mixed with PEI (1:8) or ExpifectamineTM (1:2.7) for a 15-min incubation. The mixtures were inoculated with cells and various supplements for expression enhancements were added 16 hr post-transfection. After a five-day-culturing at 37 °C, conditioned media were harvested and filtered with 0.2 µm for purification.

### 4.2. Protein Purification

As described previously [70], a 5 mL HiTrap MabSelect SuRe column (Cytiva, Marlborough, MA, USA) was pre-equilibrated with 50 mM Tris, 150 mM NaCl, pH 7.5 (TBS), and loaded with conditioned media at a flowrate of 1.25 mL/min. After the loading was completed, the column was washed with 2 column volumes (CV) of TBS, 5 CVs of 0.5 M CaCl_2_, pH 7.5, 3 CVs of 10 mM Tris, 10 mM NaCl, pH 7.5. The bound antibody was eluted using 100% step of 150 mM Glycine, 40 mM NaCl, pH 3.5, detected by monitoring absorption at 280 nm. The pH of the eluted protein sample was adjusted to 7.0 using 2 M HEPES, pH 8.0. The protein was loaded onto a Superdex 200 column (Cytiva, Marlborough, MA, USA) equilibrated with 8.1 mM Na_2_HPO_4_, 1.47 mM KH2PO4, 150 mM NaCl, 2.7 mM KCl pH 7.2 (PBS). Peak fractions were pooled and then concentrated to 10 mg/mL using a 50 kDa MWCO centrifugal device.

### 4.3. Anion-Exchange (AEX) Chromatography

Analytical strong anion-exchange chromatography (SAX) was performed using an Agilent Infinity 1290 UPLC (Agilent, Santa Clara, CA, USA) fitted with a Q-STAT (Tosoh Bioscience, South San Francisco, CA, USA), and approximately 20 to 30 ug of protein was injected at a flow rate of 1 mL/min onto the column equilibrated in 20 mM Tris pH 8.6. The protein was then eluted with 1 M NaCl in 20 mM Tris pH 8.6 (buffer B) over a 7-min linear gradient from 0–100%. Protein was detected by absorption at 280 nm. Preparative AEX was performed using an AKTA Avant 25 (Cytiva, Marlborough, MA, USA) fitted with a Mono S column (Cytiva, Marlborough, MA, USA) equilibrated in 20 mM Tris pH 8.6. The protein was then eluted with 1 M NaCl in 20 mM Tris pH 8.6 (Buffer B) over a 20 CV linear gradient from 0–100% buffer B.

### 4.4. Liquid Chromatography Mass Spectrometry (LC–MS)

As described previously [70], liquid chromatography mass spectrometry analysis was performed using a Waters Xevo Q-TOF G2 mass spectrometer (Waters, Milford, MA, USA) coupled to an Agilent (Santa Clara, CA, USA) 1200 capillary HPLC. Protein samples were treated with PNGase F (New England BioLabs, Ipswich, MA, USA) at room temperature for 2 h. Subsequent incubation with LysC (Genovis Inc., Cambridge, MA, USA) was also performed to separate Fab_2_ fragment from the single-chain fragment crystallizable (scFc). Protein samples were acidified by diluting 1:1 with 0.05% trifluoroacetic acid (TFA, Sigma-Aldrich, St. Louis, MO, USA), followed by the LC–MS analysis. The samples were separated over a Waters BEH300 C4, 1.7 µm (1.0 × 50 mm) column maintained at 80 °C with a flow rate of 65 µL/min. Mobile phase A was water with 0.05% TFA, and mobile phase B was acetonitrile with 0.05% TFA. Proteins were eluted from the column using a gradient: 2% to 20% B in 0.5 min, 20% to 40% B in 6 min, and 40% to 100% B in 4 min. The mass spectrometer was run in positive-MS-only mode scanning from 800 to 3500 m/z and data were acquired with MassLynx (Waters, Milford, MA, USA) 4.1 software. The TOF-MS signal corresponding to the antibody were summarized and deconvoluted using MaxEnt1 (Waters, Milford, MA, USA) program.

For intact and reduced antibody LC–MS analyses, protein samples were deglycosylated with PNGase F for 2 h at 37 °C for intact analysis. For reduced analysis, after deglycosylation, protein samples were reduced with 50 mM DTT (Thermo Fisher Scientific, Waltham, MA, USA) in the presence of 5 M guanidine HCl (Thermo Fisher Scientific, Waltham, MA, USA) for 1 h at 37 °C. The LC–MS analysis was performed using an ultrahigh-resolution ESI-QTOF Bruker maXis II mass spectrometer coupled to a Waters H-Class UPLC. The samples were injected onto a Waters BioResolve RP mAb Polyphenyl column (450 Å, 2.7 µm, 2.1 × 150 mm) maintained at 65 °C with a flow rate of 400 µL/min. Mobile phase A was 0.1% TFA in water (*w*/*v*), mobile phase B was 0.1% TFA in acetonitrile (*w*/*v*). For intact analysis, proteins were eluted from the column to the mass spectrometer using a linear gradient of 20% B increasing to 85% B over 5 min. For reduced analysis, proteins were eluted from the column to the mass spectrometer using a linear gradient of 20% B increasing to 57% B over 40 min. The mass spectrometer was run in positive-MS-only mode scanning from 850 to 4000 *m*/*z* and data were acquired with QTOF control software. The QTOF–MS signal was deconvoluted using Maximum Entropy in Compass Data Analysis v 4.4 (Bruker, Billerica, MA, USA).

### 4.5. Biacore Characterization

To characterize binding kinetics by surface plasmon resonance, anti-human IgG (Cytiva, Marlborough, MA, USA) was directly immobilized via amine coupling onto a carboxymethylated dextran sensor chip surface (CM5) at a density of 4000 to 6000 resonance units (RU) using a Biacore T200 instrument. For each binding cycle, anti-IL-4 was captured on the anti-IgG followed by IL-4 binding or buffer and regeneration of the IgG surface. Each Pfizer human anti-IL-4 clone was diluted to 2 µg/mL in PBS, 3.4 mM EDTA and 0.01% tween 20 (PBS-NET), and injected onto the anti-hIgG surface for 1 min at a flow rate of 5 μL/minute to achieve captured levels of approximately 75–100 RU. After each capture, the flow rate was increased to 100 μL/min and various concentrations of recombinant, carrier-free human IL-4 (R&D Systems, Minneapolis, MN, USA, Catalog 204-IL/CF) ranging from 0.195 nM to 100 nM in PBS-NET were injected for a 3-min association and allowed to dissociate for 10–60 min. At the end of each cycle, the entire anti-hIgG surface was regenerated by a 30-s pulse of 3 M MgCl2 followed by two consecutive 15-s pulses of PBS-NET. All injections were performed at 25 °C at a collection rate of 10 Hz. All sensorgrams were double referenced by using both a control surface and buffer injections. Data were collected from six flow cells of immobilized IgG in two experiments. Rate constants were determined by fitting the data to a 1:1 model in Biacore T200 Evaluation Software v1.0 and the equation KD = kd/ka. Theoretical Rmax was calculated as follows: molecular weight of analyte/molecular weight of ligand x captured level of ligand (response units) x apparent stoichiometry.

### 4.6. STAT6 Phosphorylation Assay

IL 4Rα/IL 13α1 expressing HT 29 human colonic epithelial cells (ATCC, Manassas, VA, USA) were grown as an adherent monolayer in McCoy’s 5A medium containing 10% FBS, 50 U/mL penicillin, 50 μg/mL streptomycin, and 2 mM L glutamine. For assay, the cells were dislodged from the flask using trypsin, washed into fresh medium, and distributed into 96-well cluster tubes (VWR, Bridgeport, NJ, USA). Dilutions of antibody ranging from 20 nM 0.001 nM were added, along with 0.3 ng/mL recombinant human IL-4 (R&D Systems. Cells were incubated in a 37 °C water bath for 30 min, fixed in 1.75% formaldehyde in PBS, washed with PBS containing 0.5% BSA, and incubated overnight at 20 °C in absolute methanol to permeabilize the nucleus. Fixed cells were stained with PE-labeled antibody to human pSTAT6 (pY641, Clone 18; BD Biosciences, Franklin Lakes, NJ, USA). Fluorescence was analyzed with a FACSCalibur equipped with a plate-based autosampler (BD Biosciences, Franklin Lakes, NJ, USA). Data were analyzed using FlowJo software (Tree Star Inc., Ashland, OR, USA).

### 4.7. Structure Modeling

The structure of 3B9 in complex with IL-4 was solved using X-ray crystallography (unpublished data) in the buffer conditions containing 2M ammonium sulfate or 200 mM sodium sulfate. A molecular dynamics simulation for 3B9 antibody in complex with IL14 was prepared using the Protein Preparation tool in Maestro 10 (Schrodinger, LLC, New York, NY, USA). A buffer sulfate near the Tyr 31 (Kabat L27D) side chain was removed and a covalently bound sulfate was modeled onto the Tyr 31 (L27D) side chain. A solvated orthorhombic box with 15Å buffers on each side was generated. The system was relaxed/equilibrated (using the default protocol) and then simulated for 250 ns using the OPLS4 force field in Desmond release 2023-3 (Schrodinger, LLC). Distances between the sulfate sulfur and either the guanidinium carbon (Arg81,85,88) or CE carbon (Lys84) in IL-4 were monitored during the 250 ns simulation.

## Figures and Tables

**Table 1:** Summary of IC50 values for the anti-IL-4 antibody 3B9 species in neutralization of IL-4 induced pSTAT6 formation in HT-29 cells.

Sample Name	IC50 ^1^
3B9 WT	0.12 nM
3B9 P1	0.14 nM
3B9 P2	0.046 nM
3B9 P3	0.030 nM
3B9 + TPST-1/TPST-2	0.035 nM
3B9 + NaCIO3	0.17 nM

^1^ The assays were performed as described in Section 4. For this, “3B9 + TPST-1/TPST-2” was purified from TPST-1/TPST-2-cotransfected cell culture and “3B9 + NaClO_3_” was purified from chlorate-treated cell culture.

## Data Availability

All data are published in this article.
